# Erythropoietin prevents PC12 cells from beta-amyloid-induced apoptosis via PI3K⁄Akt pathway

**DOI:** 10.1186/2047-9158-1-7

**Published:** 2012-02-29

**Authors:** Sun Zhi-Kun, Yang Hong-Qi, Wang Zhi-Quan, Pan Jing, Hong Zhen, Chen Sheng-Di

**Affiliations:** 1 Department of Neurology & Institute of Neurology, Ruijin Hospital, Shanghai Jiaotong University School of Medicine, Shanghai 200025, People's Republic of China; 2 Lab of Neurodegenerative Diseases, Institute of Health Science, Shanghai Institutes for Biological Sciences (SIBS), Chinese Academy of Sciences (CAS) & Shanghai Jiaotong University School of Medicine, Shanghai 200025, People's Republic of China; 3 Department of Neurology & Institute of Neurology, Henan Provincial Hospital, Zhengzhou City, Henan province 450000, People's Republic of China

**Keywords:** Beta-amyloid peptide, Apoptosis, Erythropoietin, Alzheimer's disease

## Abstract

**Background:**

Several studies indicated that **Erythropoietin **(Epo) may provide remarkable neuroprotection in some neurological diseases. It also showed the significant decrease of Epo immunoreactivity in the cerebral cortex and hippocampus in aged rats, suggesting the role of Epo in the pathogenesis of age-related neurodegenerative diseases such as AD.

**Methods:**

The protective effect of Epo was studied in differentiated PC12 cells treated with Abeta. The viability of the cells, the apoptosis of the cells and the level of Bax, Bcl-2, cleaved caspase-3 and cleaved PARP expression were detected by MTT, Hoechst 33258 staining and Western blotting respectively.

**Results:**

20 μM Abeta _(25-35) _could induce a decreased viability and a increased apoptosis in PC12 cell in a time-dependent manner. However, 20 μM Abeta _(35-25) _had no effect on cell viability and apoptosis. Western blot analysis also showed that Abeta_(25-35) _treatment could decrease the expression of Bcl-2 (*P *< 0.05) and increase the expression of Bax (*P *< 0.05), Cleaved casapase-3 (*P *< 0.05), and Cleaved PARP (*P *< 0.05). The pretreatment of Epo could effectively reverse all the above changes induced by Abeta_(25-35_) (*P *< 0.05). Furthermore, the protective effect of Epo could be blocked by PI3K inhibitor LY294002 (*P *< 0.05).

**Conclusions:**

Epo prevented cell injuries in PC12 cells exposed to the Abeta_(25-35) _and this effect may depend on the PI3K⁄Akt pathway. Our study provided an important evidence for the potential application of Epo in the therapy of Alzheimer's disease.

## Background

Apoptosis is a particular type of programmed cell death controlled by precise intrinsic genetic programme in order to regulate cell population. Among the mechanisms of cell death, apoptosis has been proposed to explain the cell loss observed in many neurodegenerative disorders including Alzheimer's disease (AD) [1-3]. AD is a neurodegenerative disorder of the central nervous system (CNS), which correlate with the appearance of neurofibrillary tangles (NFTs) and senile plaques (SPs) [4]. The major component of SPs is beta-amyloid peptide (Abeta), which is believed to be the most probable cause of AD [3,5]. Many studies have shown that Abeta can directly induce neuronal death via apoptosis [2,6,7].

Erythropoietin (Epo) was originally characterized as the principal regulator of erythropoiesis [8]. Many experimental studies have shown that both Epo and its specific receptor (erythropoietin receptor, EpoR) expressing in the CNS, provide remarkable neuroprotection in many neurological diseases [9-13]. Recent research has demonstrated significant decreases in Epo immunoreactivity in the cerebral cortex and hippocampus of aged rats [14] which suggested the role of Epo in the pathogenesis of age-related neurodegenerative diseases such as AD. Therefore, we studied the possible relationship between Epo and Abeta-induced cell apoptosis. In the present study, we observed that Abeta_(25-35) _peptide at 20-μM concentrations could induce apoptosis in PC12 cells and Epo could reverse these changes through PI3K/Akt signaling pathway. Our results identifed a potential molecular targets for AD therapy.

## Materials and methods

### Cell culture and drug treatment

Abeta_(25-35) _(Sigma-Aldrich, St. Louis, MO) or Abeta_(35-25) _(Sigma-Aldrich, St. Louis, MO) was dissolved in water to obtain a 2 mM stock solution. Aliquots were stored at -20°C and thawed at 37°C for 5 ~ 7 d for use. Differentiated rat pheochromocytoma PC12 cells (provided by the Institute of Biochemistry and Cell Biology, Chinese Academy of Science, Shanghai) were plated in 100-mm culture dishes (Corning Incorporated, Corning, NY, USA) in DMEM containing 10% (v/v) heat-inactivated FBS, 5% horse serum, 1% penicillin, and 1% streptomycin. The cells were grown at 37°C in a humid 5% CO_2 _environment, and the medium was routinely replaced every 2 d. The media were replaced with serum-free media 12 h prior to drug treatment. The cells were then treated with Abeta_(25-35) _or Abeta_(35-25) _for 24 h. Epo (R&D systems, USA) at various concentrations were added into the cultures 1 h prior to the 24-h Abeta_(25-35) _exposure. 20 μM LY294002 (Sigma-Aldrich, St. Louis, MO, dissolved in DMSO) were added into the cultures 1 h prior to the Epo treatment.

### Analysis of cell viability

Cell viability was assessed by MTT assay. Briefly, PC12 cells were seeded in 96-well culture plates at a density of 1 × 10^4 ^cells per well. After the treatment of Abeta_(25-35)_, Abeta_(35-25)_, Epo or LY294002, the cells were subjected to the assay as previously reported [15,16].

### Hoechst 33258 staining

For Hoechst 33258 staining, cells were fixed with 4% paraformaldehyde. Cell nuclei were stained with fluorescent dye Hoechst 33258 (Sigma, St. Louis, MO) at a final concentration of 5 μg/ml in PBS, for 20 min at room temperature in a dark chamber, and then observed in a fluorescence microscope (OLYMPUS 1 × 70, Japan) and photographed.

### Western blotting

The Western blotting analysis procedure was conducted as previously reported [16]. After the treatment, cells were washed twice with cold phosphate buffered saline and lysed on ice with cell lysis buffer(10 mM Tris, pH 7.4, 100 mM NaCl, 1 mM EDTA, 1 mM EGTA, 1 mM NaF, 20 mM Na4P2O7, 2 mM Na3VO4, 0.1% SDS, 0.5% sodium deoxycholate, 1% Triton-X 100, 10% glycerol, 1 mM PMSF (made from a 0.3 M stock in DMSO), 60 μg/mL aprotinin, 10 μg/mL leupeptin, 1 μg/mL pepstatin) for 30 mininutes. The soluble fraction was obtained by centrifugation at 14000 g for 20 min at 4°C. The concentration of the protein was determined by the BCA assay (Pierce Biotechnology, Rockford, IL). Equal amounts of the protein (20 μg) were separated in an 8-10% SDS-polyacrylamide gel; the resolved proteins were electrotransferred onto PVDF or nitrocellulose membranes (Bio-Rad, Hercules, CA). The membranes were subsequently blocked with 5% nonfat milk in TBST for 1 h at room temperature and incubated with appropriate concentrations of primary antibody (1:200 for Bax and Bcl-2 (Santa Cruz Biotechnology, Inc, CA, USA), 1:5000 for beta-actin (Sigma-Aldrich, St. Louis, MO), 1:1000 for Cleaved caspase-3 and PARP (Cell Signaling Technology, Beverly, MA)) at 4°C overnight. The membranes were then washed 3 times with TBST and probed with the corresponding secondary antibodies conjugated with HRP (Cell Signaling Technology, Beverly, MA) at room temperature for 1 h. After washing, the signals were developed using the ECL Advanced Western Blotting Detection kit (Amersham, UK). Band intensities were quantified by densitometric analysis by using an AxioCam digital camera (ZEISS, Germany) and the KS400 photo analysis system (Ver. 3.0).

### Statistics

Data are expressed as mean ± standard deviation (S.D.) and were analyzed using SPSS 11.0 statistical software (SPSS Inc., Chicago, IL, USA). Each procedure was performed in duplicate in 3 ~ 5 independent experiments. Statistical analyses were performed using one-way ANOVA, followed by the two-tailed Student's *t *test. Multiple comparison tests were applied when appropriate, and statistical significance was assumed at *P *< 0.05.

## Results

### Effects of Abeta_(25-35) _on cell viability and cell apoptosis determined by MTT and Hoechst 33258 staining respectively

**Figure 1:**
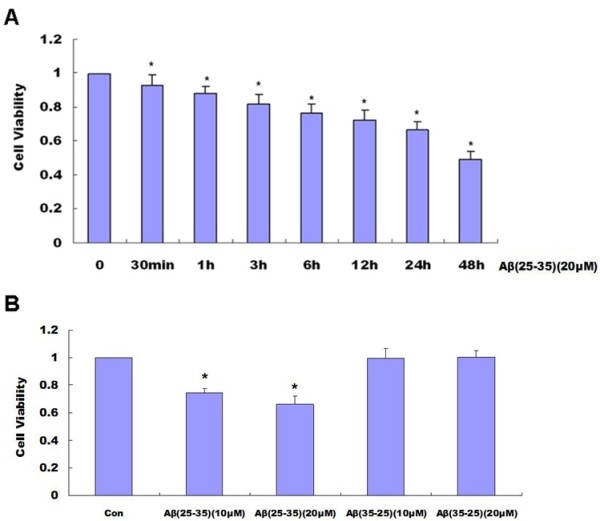
**Effects of Abeta_(25-35) _on cell viability**. The MTT assay was used to determine the cell viability. As shown in the following graph, 20 μM Abeta_(25-35) _induced a decrease in PC12 cell viability in a time-dependent manner (*P *< 0.05) **(A)**, However, 20 μM Abeta_(35-25) _had no effect on PC12 cell viability (*P *> 0.05) **(B)**. (*P < 0.05 vs. the controls). The data shown represent 5 independent experiments

**Figure 2:**
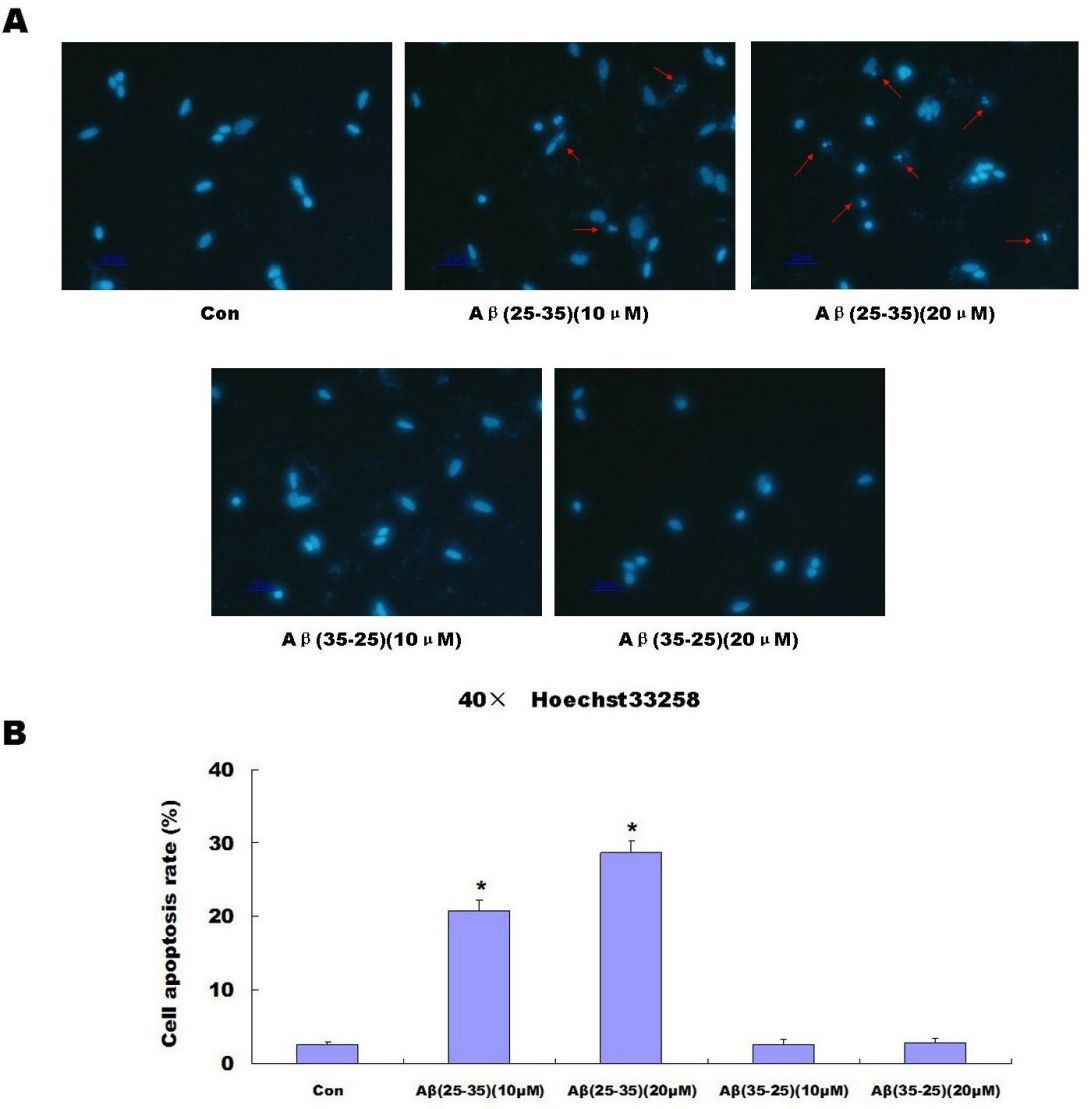
**Effects of Abeta_(25-35) _on cell apoptosis**. Hoechst33258 was performed to detect the cell apoptosis (Con, Control group), showing nuclear condensation and fragmentation (arrows) **(A)**. 10 randomized representative fields were analyzed in one experiment. 10 μM and 20 μM Abeta_(25-35) _could effectively induce PC12 cell apoptosis (*P *< 0.05) **(A and B)**, and 20 μM Abeta_(35-25) _had no effect on PC12 cell apoptosis **(A and B)**. The data shown represent 5 independent experiments (*P < 0.05 vs. the controls) **(B)**

The MTT assay was used to determine the effect of 20 μM Abeta _(25-35) _on the viability of the PC12 cell cultures. As shown in the following graph, 20 μM Abeta _(25-35) _induced a decrease in PC12 cell viability in a time-dependent manner (Figure 1A). We also used the control peptide 20 μM Abeta_(35-25) _to determine the effect of 20 μM Abeta_(35-25) _on the cell viability As shown in the following graph, 20 μM Abeta _(35-25) _had no effect on PC12 cell viability (Figure 1B). Hoechst 33258 staining also showed 10 μM Abeta _(25-35) _and 20 μM Abeta_(25-35) _could induce PC12 cell apoptosis. However, 10 μM Abeta _(35-25) _and 20 μM Abeta _(35-25) _had no effect on PC12 cell apoptosis (Figure 2A and 2B).

### Effects of Epo on Abeta_(25-35)_-induced PC12 cell viability and cell apoptosis determined by MTT and Hoechst 33258 staining respectively

**Figure 3:**
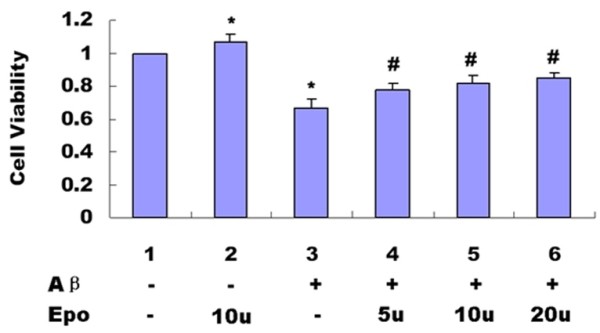
**Effect of Epo on cell viability induced by 20 μM Abeta_(25-35)_**. The MTT assay was used to determine the cell viability. As shown in the following graph, Various concentrations of Epo (5, 10, 20 u) could effectively prevent a decrease of cell viability induced by 20 μM Abeta_(25-35) _(*P *< 0.05) (B). (*P < 0.05 vs. the controls). The data shown represent 5 independent experiments

**Figure 4:**
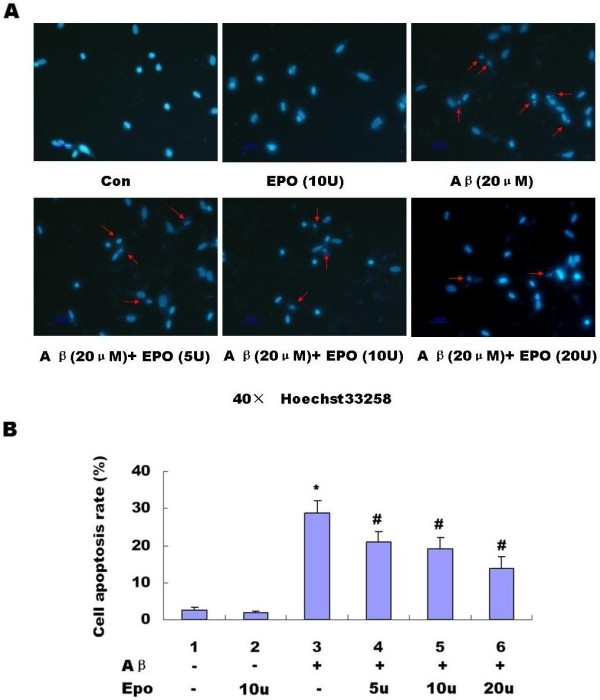
**Effect of Epo on cell apoptosis induced by 20 μM Abeta_(25-35)_**. Hoechst33258 was performed to detect the cell apoptosis (Con, Control group). We found 3 different concentrations of Epo (5, 10, 20 u) could effectively prevent cell apoptosis induced by Abeta_(25-35) _(*P *< 0.05) **(A and B)**. The data shown represent 5 independent experiments (*P < 0.05 vs. the controls) **(B)**

We added 3 different concentrations of Epo (5, 10, 20 u) into the serum-deprived media of PC12 cells 1 h prior to the 24-h 20 μM Abeta _(25-35) _exposure. As shown in the following graph, various concentrations of Epo (5, 10, 20 u) could effectively prevent a decrease of cell viability induced by 20 μM Abeta _(25-35) _(*P *< 0.05) (Figure 3). Hoechst 33258 staining also showed 3 different concentrations of Epo (5, 10, 20 u) can effectively prevent cell apoptosis induced by Abeta _(25-35) _(*P *< 0.05) (Figure 4A and 4B).

### Effects of Epo on Abeta_(25-35)_-induced PC12 cell apoptosis determined by Western blotting

**Figure 5:**
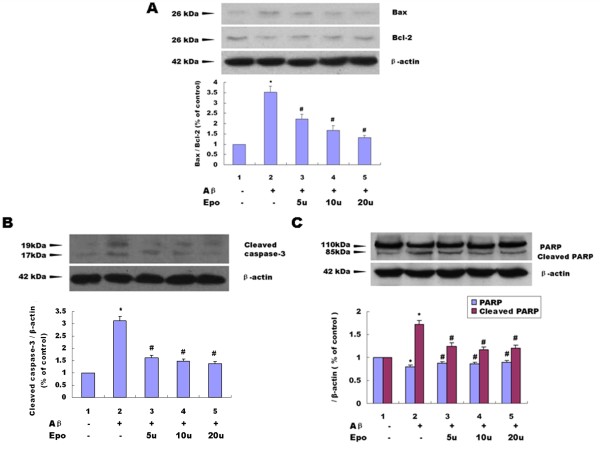
**Effect of Epo on 20 μM Abeta_(25-35)_-induced cell apoptosis**. Western blotting analysis indicated that the Abeta_(25-35) _treatment of PC12 cells could decrease the expression of Bcl-2 (*P *< 0.05) **(A)**, and increase the expression of Bax (*P *< 0.05) **(A)**, Cleaved casapase-3 (*P *< 0.05) **(B)**, and Cleaved PARP (*P *< 0.05) **(C)**. Three different Epo concentrations could prevent all the changes induced by Abeta_(25-35) _(*P *< 0.05) **(A-C)**. Three independent experiments were performed in duplicate (*: *P *< 0.05 vs. the controls and #: *P *< 0.05 vs. 20 μM Abeta_(25-35)_)

Using Western blotting analysis, we found that the Abeta_(25-35) _treatment of PC12 cells could decrease the expression of Bcl-2 (*P *< 0.05) (Figure 5A) and increase the expression of Bax (*P *< 0.05) (Figure 5A), Cleaved casapase-3 (*P *< 0.05) (Figure 5B), and Cleaved PARP (*P *< 0.05) (Figure 5C). Three different Epo concentrations can prevent all the above changes induced by Abeta_(25-35) _(*P *< 0.05) (Figure 5A-C).

### PI3K/Akt involvement in the effects of Epo on Abeta _(25-35)_-induced cell injuries

**Figure 6:**
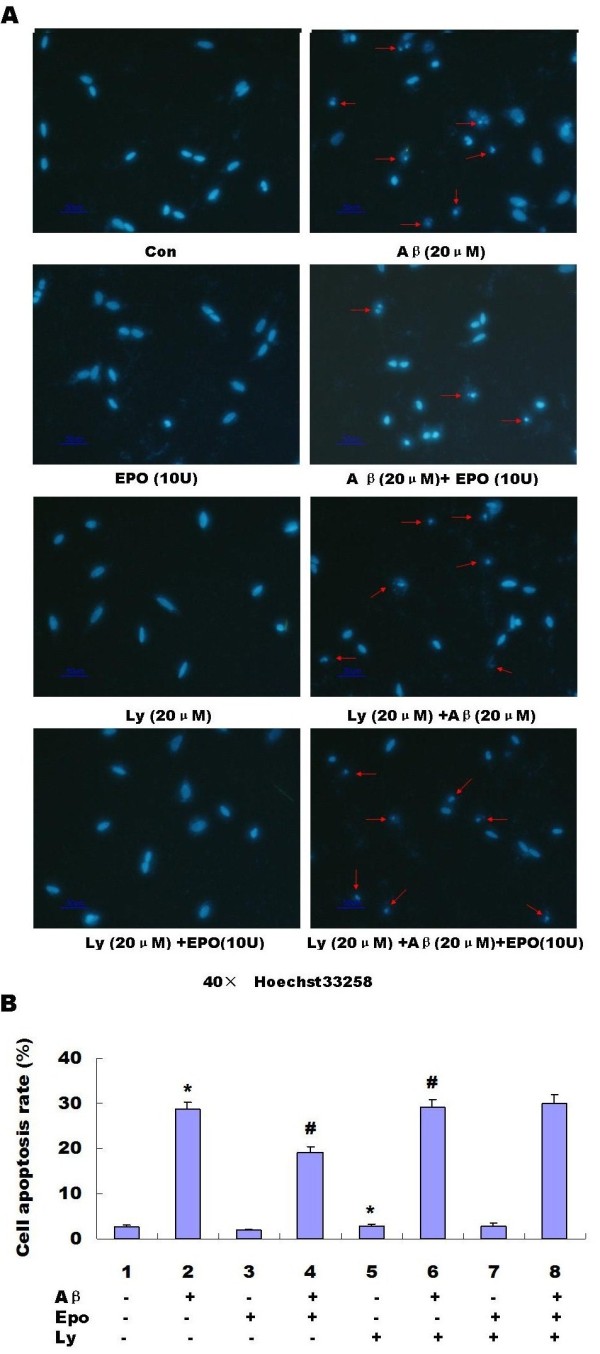
**Involvement of PI3K/Akt in the effects of Epo on Abeta _(25-35)_-induced cell apoptosis determined by Hoechst33258 staining**. As shown in the following graph, We treated the cells with PI3K inhibitor LY294002 and found the protective effects of Epo on the Abeta_(25-35)_-induced cell apoptosis were diminished (*P *< 0.05). Three independent experiments were performed in duplicate (*: *P *< 0.05 vs. the controls and #: *P *< 0.05 vs. 20 μM Abeta_(25-35)_)

**Figure 7:**
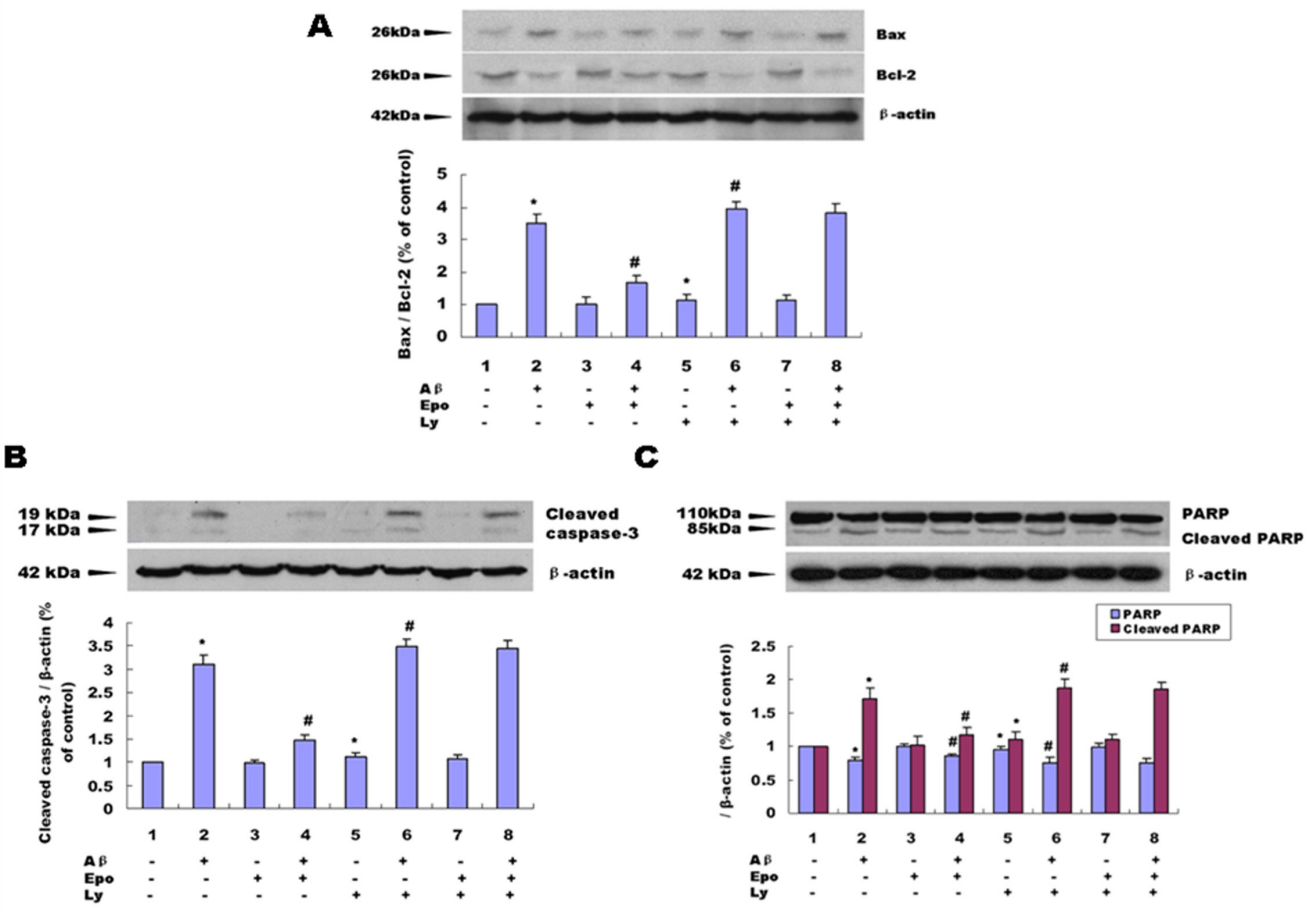
**Involvement of PI3K/Akt in the effects of Epo on Abeta _(25-35)_-induced cell apoptosis determined by Western blotting analysis**. As shown in the graph, We treated the cells with PI3K inhibitor LY294002 and found the protective effects of Epo on Abeta_(25-35)_-induced cell apoptosis were diminished (*P *< 0.05) **(A-C)**. Three independent experiments were performed in duplicate (*: *P *< 0.05 vs. the controls and #: *P *< 0.05 vs. 20 μM Abeta_(25-35)_)

**Figure 8:**
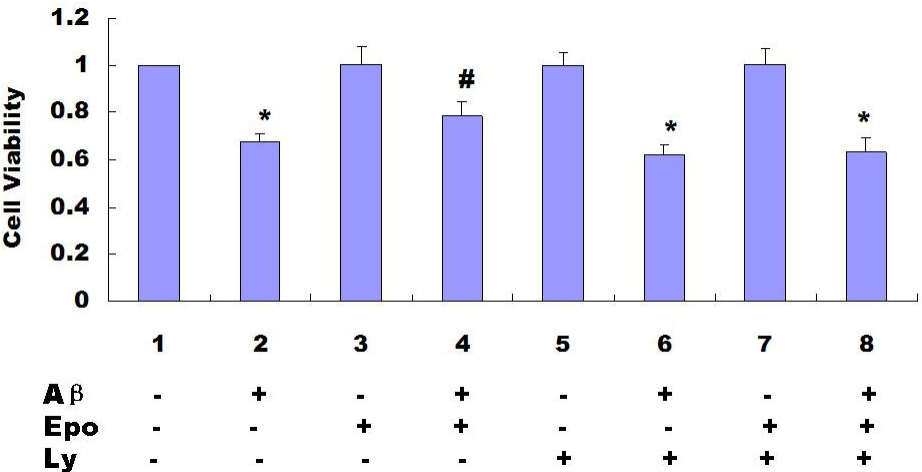
**Involvement of PI3K/Akt in the effects of Epo on the Abeta_(25-35)_- decreased cell viability determined by MTT**. We treated the cells with PI3K inhibitor LY294002 and found the protective effects of Epo on the Abeta_(25-35)_-decreased cell viability were diminished (*P *< 0.05). Three independent experiments were performed in duplicate (*: *P *< 0.05 vs. the controls and #: *P *< 0.05 vs. 20 μM Abeta_(25-35)_).

Stimulation of EpoRs by Epo has previously been shown to activate the PI3K⁄Akt signal transduction pathway [17,18], which regulates cell survival and proliferation [19]. We treated the cells with PI3K inhibitor LY294002 and found the LY294002 treatment caused a slight increase in cell apoptosis in PC12 cells with or without Abeta_(25-35) _treatment (Figure 6A, B and Figure 7A-C) This suggested that the PI3K/Akt pathway was involved in Abeta_(25-35)_-induced cell apoptosis, When the PI3K pathway was inhibited by LY294002 in PC12 cells, we found that the effects of Epo on Abeta_(25-35)_-induced cell injuries were diminished (*P *< 0.05) (Figure 8, Figure 6A, B and Figure 7A-C).

## Discussion

Abeta is the major component of SPs, which are considered to play a causal role in the development and progress of AD [20,21]. The molecular mechanisms underlying Abeta-mediated neurotoxicity remain unclear. Recently, many in vitro and vivo studies have shown that Abeta can directly induce neuronal death via the mechanism of apoptosis [2,3,22]. Epo is widely known for its role as a hematopoetic hormone. Epo binds to specific receptors present in the human brain can be synthesized by astrocytes as well as neurons [23]. Epo was shown to be capable of crossing the blood-CSF barrier via receptor-mediated transport [24,25] and to act as a neurotrophic factor supporting the differentiation and regeneration of neurons [26]. Its protective effect under conditions of neuronal injury was also reported [27,28]. Therefore, we proposed that the Epo system in the CNS can act as an endogenous system for protecting against neurodegenerative diseases such as AD. Among the fragments studied so far, the Abeta_(25-35) _represents the shortest fragment of Abeta, processed in vivo by brain proteases [29]. This peptide is the functional domain of Abeta required for neurotoxic effect, retaining the toxicity of the full-length peptide [30,31]. It is highly cytotoxic to neuronal cells [32-34] and is widely used in both in vitro and in vivo experiments [35,36]. In the present study, we used Abeta_(25-35) _to observe the toxic effect of Abeta and the protective effect of Epo. Abeta_(35-25)_, a 11 amino acid with a reverse sequence of Abeta_(25-35) _was used as a control. We discovered that aggregated 20 μM Abeta_(25-35) _could decrease cell viability in a time-dependent manner (Figure 1A), However, 20 μM Abeta _(35-25) _had no effect on PC12 cell viability (Figure 1B.). Hoechst 33258 staining showed Abeta_(25-35) _can induce PC12 cell apoptosis while Abeta_(35-25) _had no effect on PC12 cell apoptosis (Figure 2A and 2B). Epo could attenuate the decreased cell viability (Figure 3) and increased cell apoptosis (Figure 4A and 4B) induced by Abeta_(25-35)_.

Apoptosis is a tightly regulated process which involves changes in the expression of a distinct set of genes [37]. Bcl-2 is a key member of the anti-apoptotic Bcl-2 family, which plays a key role in regulating mitochondrial-mediated apoptotic cell death [38-40]. Over-expression of Bcl-2 can protect neuronal cells from neurotoxic insult. In contrast, Bax belongs to the pro-survival subfamily, which promotes apoptosis by translocating into the mitochondrial membrane and facilitating cytochrome c release [41]. In the present study, we found 20 μM Abeta_(25-35) _exposure could induce an increase of Bax expression and decrease Bcl-2 expression in serum-deprived cultured PC12 cells (Figure 5A), and Epo could effectively attenuate these changes (Figure 5A).

Caspases are a family of cysteine proteases and are critical mediators of cell apoptosis, which play an important role in the apoptotic process [42]. Caspase-3 acts as an apoptotic executor, it can activate DNA fragmentation factor, which in turn activate endonucleases to cleave nuclear DNA, and ultimately leads to cell death [43,44]. Activation of caspase-3 appears to be a key event in execution of the apoptotic cascade in CNS diseases such as AD and Down's syndrome [45,46]. In this study, we also found 20 μM Abeta_(25-35) _exposure could induce an increase of Cleaved caspase-3 expression (Figure 5B), and Epo could effectively attenuate these changes (Figure 5B).

Significant evidence indicates that caspase-3 is either partially or totally responsible for the proteolytic cleavage of many key proteins, including PARP. PARP is a nuclear DNA-binding protein of 110 kDa that is constitutively expressed in eukaryotes and that comprises up to 1% of the total nuclear proteins [47,48]. PARP is important for cell viability, and cleavage of PARP facilitates cellular disassembly and serves as a marker of cells undergoing apoptosis [49]. In this study, we also found 20 μM Abeta_(25-35) _exposure could induce an increase of Cleaved PARP expression and Epo could effectively attenuate these changes (Figure 5C) with the same trend as the expression of Cleaved caspase-3 (Figure 5B).

Epo elicits its effects by binding to specific cell surface receptors. Evidence shows that Epo can induce activation of JAK-2/STAT-5 [50,51], PI3K/Akt kinase [19], MAPK [52,53], and PKC [54]. In the present study, we examined the effects of Epo on Abeta_(25-35)_-induced cell apoptosis in PC12 cells. We found Abeta_(25-35)_-mediated cell apoptosis could be appropriately attenuated by Epo (Figure 5A-C). Further, we found that LY294002, a PI3K inhibitor, attenuated the effect of Epo on Abeta_(25-35)_-induced-cell injuries (Figures 8, 6, 7), indicating that the protective effect of Epo is dependent on PI3K signaling. Our findings provide new molecular insight into the neuroprotective effect of Epo and suggest its possible therapeutic role in the management of AD.

## Conclusions

In this report, we report that Epo prevented cell injuries in PC12 cells exposed to the beta-amyloid peptide and that this effect may depend on the PI3K⁄Akt pathway. The present study provides new molecular insight into the neuroprotective effect of Epo and suggests its possible therapeutic role in the management of AD.

## Abbreviations

AD: Alzheimer's disease; DMSO: Dimethyl sulphoxide; Epo: Erythropoietin; EpoR: Erythropoietin receptor; FBS: Foetal bovine serum; MAPK: Migoten activated protein kinase; PARP: Poly (ADP-ribose) polymerase; PI3K: Phosphorinositide 3-kinase; PVDF: Polyvinylidene difluoride

## Competing interests

The authors declare that they have no competing interests.

## Authors' contributions

Z-KS and H-QY made substantial contributions to conception and design, acquisition of data, and analysis and involved in drafting the manuscript. Z-QW and JP participated in the design of the study and performed the statistical analysis. ZH made interpretation of data and involved in revising it critically for important intellectual content. S-DC were the general supervision of the research group, acquisition of funding, and involved in revising it critically for important intellectual content. All authors read and approved the final manuscript.
